# A new post-synthetic route to graft amino groups in porous organic polymers for CO_2_ capture†

**DOI:** 10.1039/d5sc00355e

**Published:** 2025-07-01

**Authors:** Qihaoyue Wang, Lin Lin, Li Jiang, Zihao Wang, Yina Zhang, Qiance Han, Xin Huang, Changyan Zhu, Jiangtao Jia, Zheng Bian, Guangshan Zhu

**Affiliations:** a Key Laboratory of Polyoxometalate and Reticular Material Chemistry of Ministry of Education, Faculty of Chemistry, Northeast Normal University Changchun Jilin 130024 China jiangtaojia@nenu.edu.cn bianz070@nenu.edu.cn zhugs@nenu.edu.cn

## Abstract

Herein, we report the development of a post-synthetic modification approach to introduce a high loading of formyl groups onto porous aromatic framework (PAF)-5 *via* Friedel–Crafts alkylation followed by hydrolysis. Rigorous characterization by NMR, X-ray photoelectron spectroscopy, and Fourier-transform infrared spectroscopy authenticated the successful integration of aldehyde moieties into PAF-5, affording PAF-5-CHO. Subsequent functionalization of PAF-5-CHO with various amines produced three amine-functionalized PAF derivatives. Notably, PAF-5-C=N-EDA exhibited a 78% enhancement in carbon dioxide (CO_2_) adsorption capacity, reaching 3.78 mmol g^−1^ at 1 bar and 298 K relative to PAF-5-CHO. Breakthrough experiments demonstrated that PAF-5-C=N-EDA could effectively separate CO_2_ from simulated flue gas (CO_2_/N_2_ = 15 : 85, v/v; 10 mL min^−1^). *In situ* infrared spectroscopy, density functional theory calculations and temperature-programmed desorption studies provided insights into the CO_2_ adsorption mechanism.

## Abstract 2

Herein, we report the development of a post-synthetic modification approach to introduce a high loading of formyl groups onto porous aromatic framework (PAF)-5 *via* Friedel–Crafts alkylation followed by hydrolysis.

## Graphical abstract

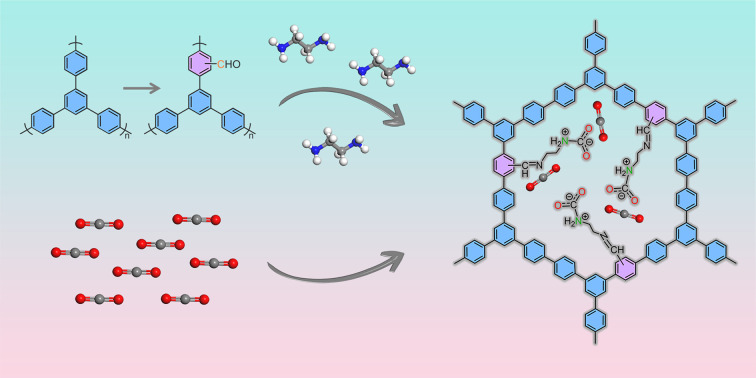

## Introduction

Since the Industrial Revolution in the 18th century, the excessive exploitation and combustion of carbon-based fossil fuels such as coal, oil, and natural gas have driven a remarkable increase in carbon dioxide (CO_2_) emissions across the globe.^1^ As a result, severe environmental issues have emerged, including the greenhouse effect, ocean acidification, sea-level rise, and climate change, all of which pose significant threats to human well-being. Replacing traditional fossil fuels with clean energy sources such as solar, hydrogen, wind, or nuclear energy represents a promising strategy for mitigating CO_2_ emissions. However, the current exploitation and implementation of clean energy fall short of meeting the established emission reduction targets.^2^ Therefore, CO_2_ capture and storage (CCS), a process that separates CO_2_ from the flue gas of coal-fired power plants and stores it underground, remains a key strategy for reducing global CO_2_ emissions during the transition to clean energy.^3^

There are five principal methods for capturing CO_2_: membrane separation, solvent-based absorption, physical adsorption, cryogenic separation, and chemical looping.^4^ Among these, aqueous amine solutions, which capture CO_2_*via* the formation of carbamates or bicarbonates, remain the most mature and widely applied technology. However, aqueous amine systems suffer from significant drawbacks, including volatility, oxidative degradation, equipment corrosion, and high operational costs.^5^ Due to these limitations, several new CCS methods have emerged in recent years, among which solid porous materials, such as activated carbons, metal–organic frameworks (MOFs), porous organic polymers (POPs), silica, and zeolites, have received considerable attention and extensive research.^6–11^ These materials offer advantages like high adsorption efficiency, low energy consumption, stability, and ease of regeneration.

Post-synthetic modification (PSM) is a vital approach for developing and enhancing solid materials such as MOFs and POPs, offering extensive functionality and broad applicability.^12–19^ Incorporating CO_2_-philic functional groups into porous materials by PSM has been proven to enhance strong interactions between CO_2_ and materials, significantly improving their selectivity for CO_2_ capture.^20–24^ Porous aromatic frameworks (PAFs), a subset of POPs, possess high specific surface areas and porosities, which significantly enhance gas contact efficiency. PAFs are typically constructed through C–C covalent bonds, and their robust skeletons confer high chemical stability, enabling them to withstand harsh conditions during the PSM process. In previous studies, PSM has been used to introduce functional groups such as –SH, –NH_2_, –SO_3_H, and –Cl into PAF frameworks, enabling their applications in areas such as seawater desalination, gas adsorption, and catalysis.^25–36^ In addition, PAFs are ideal candidates for the incorporation of CO_2_-philic functional groups, which can strongly interact with CO_2_ and thus facilitate efficient capture.

In this study, we propose a new PSM strategy to enhance the CO_2_ capture efficiency of PAFs. This method allows for the simple and efficient introduction of aldehyde groups into the PAF framework by way of the Friedel–Crafts reaction, resulting in the synthesis of PAF-5-CHO. Notably, direct PSM of –CHO groups in the frameworks has not yet been reported. The aldehyde group has the potential to be transformed into different functional groups.^37,38^ Accordingly, PAF-5-CHO was further functionalized with amino groups *via* Schiff base reactions with organic amines aiming to enhance CO_2_ capture performance. The resulting series of amino-functionalized PAFs exhibit excellent CO_2_ capture and separation properties.

## Results and discussion

### Synthesis and characterization of PAF-5-CHO

**Fig. 1:**
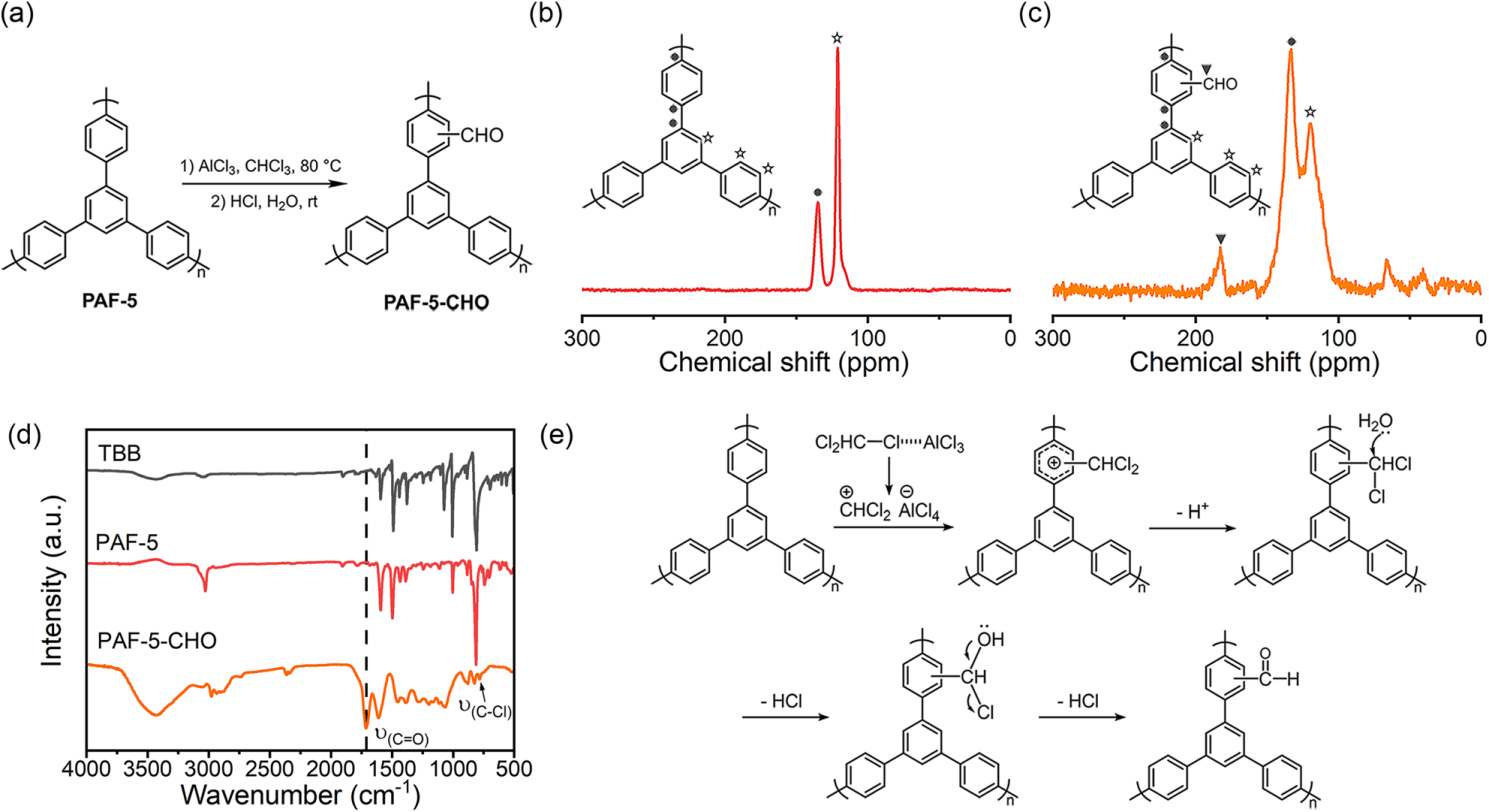
(a) The synthesis of PAF-5-CHO; (b) ^13^C CP/MAS NMR spectrum of PAF-5; (c) ^13^C CP/MAS NMR spectrum of PAF-5-CHO. (d) FTIR spectra of PAF-5-CHO, PAF-5, and TBB (1,3,5-tris(4-bromophenyl) benzene); (e) the formation mechanism of –CHO groups.

PAF-5 was synthesized as reported^39^ and the detailed synthesis method of PAF-5-CHO is described in the Experimental section. The dichloromethylation of PAF-5 in chloroform under the activation of anhydrous AlCl_3_ and subsequent hydrolysis afforded PAF-5-CHO (Fig. 1a). In the solid-state carbon ^13^C NMR spectrum of PAF-5 (Fig. 1b), the peaks at 135 ppm and 120 ppm correspond to the substituted and unsubstituted carbons on phenyl rings, respectively.^39^ Compared to PAF-5, the spectrum of PAF-5-CHO (Fig. 1c) showed similar peaks at 133 ppm and 120 ppm for the substituted and unsubstituted carbons on phenyl rings, but with a different signal at 183 ppm. According to the literature,^40^ this peak was attributed to the carbon of the aldehyde group, confirming the successful introduction of aldehyde functional groups. The signals at 40 ppm and 67 ppm were attributed to the carbon of –CHCl– and –CHCl_2_ groups,^41^ respectively, indicating that partial –CHCl_2_ groups and –CHCl– groups remained unhydrolyzed due to the steric effect or dead-end pore (Fig. S1†). Additionally, in the Fourier transform infrared (FTIR) spectrum (Fig. 1d), the peak at 1700 cm^−1^ was characteristic of the C=O bond of aldehyde groups,^23^ while the peak at 783 cm^−1^ was attributed to the unhydrolyzed C–Cl bond.^27^ The formation mechanism of aldehyde groups in PAF-5-CHO by way of Friedel–Crafts alkylation and acidic hydrolysis is illustrated in Fig. 1e. Notably, the method to introduce –CHCl_2_ groups by Friedel–Crafts alkylation is limited to the rigid organic framework. In the rigid aromatic porous material, these –CHCl_2_ groups can't further react with other aromatic units, making them completely stay on the framework.

Additionally, the high-resolution C 1s XPS spectrum of PAF-5 (Fig. S2†) shows only a single peak corresponding to C–C bonds. In contrast, the C 1s spectrum of PAF-5-CHO displays new peaks at 286.5 eV and 288.7 eV, corresponding to C–Cl and C=O bonds, respectively.^42^ Elemental oxygen analysis of PAF-5-CHO (Table S1†) reveals an oxygen content of 7.475 wt%, indicating the introduction of approximately 1.5 aldehyde groups per structural unit of PAF-5. However, thermogravimetric analysis (TGA) results (Fig. S3a†) show a weak thermal stability for PAF-5-CHO compared to PAF-5 due to the lower pyrolysis temperature of –CHO groups than aromatic groups. Furthermore, the scanning electron microscope (SEM) images (Fig. S4a–c†) exhibit no marked changes after the modification process. Powder X-ray diffraction (PXRD) patterns (Fig. S5†) indicate that PAF-5-CHO remains an amorphous material.

### Synthesis of organic amine-modified PAF-5 materials

**Fig. 2:**
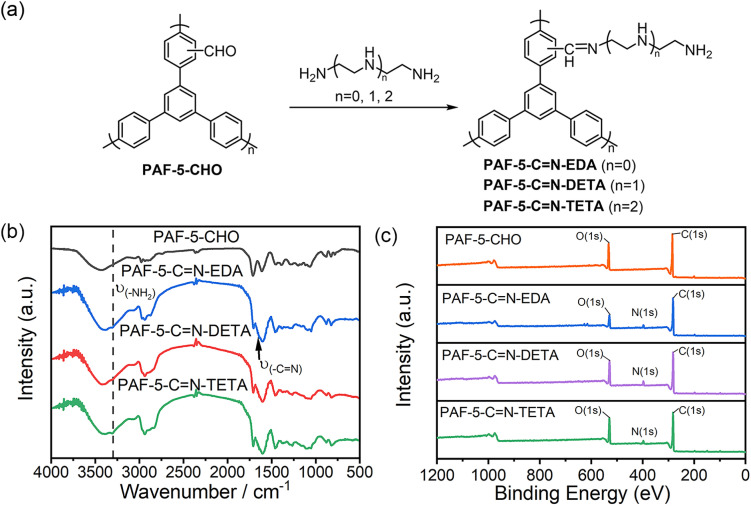
(a) Synthetic route to organic amine-modified PAF-5; (b) FTIR spectra of PAF-5-C=N-TETA, PAF-5-C=N-DETA, PAF-5-C=N-EDA and PAF-5-CHO; (c) XPS full-spectrum of PAF-5-CHO, PAF-5-C=N-TETA, PAF-5-C=N-EDA and PAF-5-C=N-DETA.

To introduce CO_2_-philic functional groups, we selected three organic amines with different chain lengths and successfully synthesized a series of amine-functionalized PAF-5 materials *via* Schiff base reactions (Fig. 2a). In the FTIR spectra of PAF-5-C=N-EDA, PAF-5-C=N-DETA, and PAF-5-C=N-TETA (Fig. 2b), the emergence of –NH_2_ vibration at 3300 cm^−1^ and –C=N vibration at 1640 cm^−1^ confirms the successful occurrence of the Schiff base reaction.^23^ In the XPS total spectrum (Fig. 2c), the presence of the N element can be clearly evident. Additionally, in the high-resolution N 1s XPS spectra of PAF-5-C=N-EDA, PAF-5-C=N-DETA, and PAF-5-C=N-TETA (Fig. S6†), the peaks at 398.6 eV, 398.3 eV, and 398.2 eV are assigned to C=N bonds, while the peaks at 399.4 eV, 399.1 eV, and 399 eV correspond to –NH_2_ and –NH– species.^43–45^ Elemental analysis (Table S2†) further supports the formation of imine bonds, showing a significant increase in nitrogen content for the three amine-functionalized materials compared to PAF-5-CHO. Among them, PAF-5-C=N-TETA exhibits the highest nitrogen content at 10.04 wt%, equivalent to approximately 0.8 -NH_2_ groups per triphenyl benzene unit of PAF-5. Based on these data, it can be inferred that around 50% of the aldehyde groups participate in the reaction. Moreover, TGA analysis (Fig. S3b†) demonstrates that the amine-functionalized PAF-5-CHO materials retain stability in air up to 235 °C. The weight loss observed between 235 °C and 520 °C is attributed to the decomposition of the amine chains.^20^ SEM images (Fig. S4d–f†) reveal no significant morphological changes in the materials after amine modification.

### Gas adsorption and desorption

**Fig. 3:**
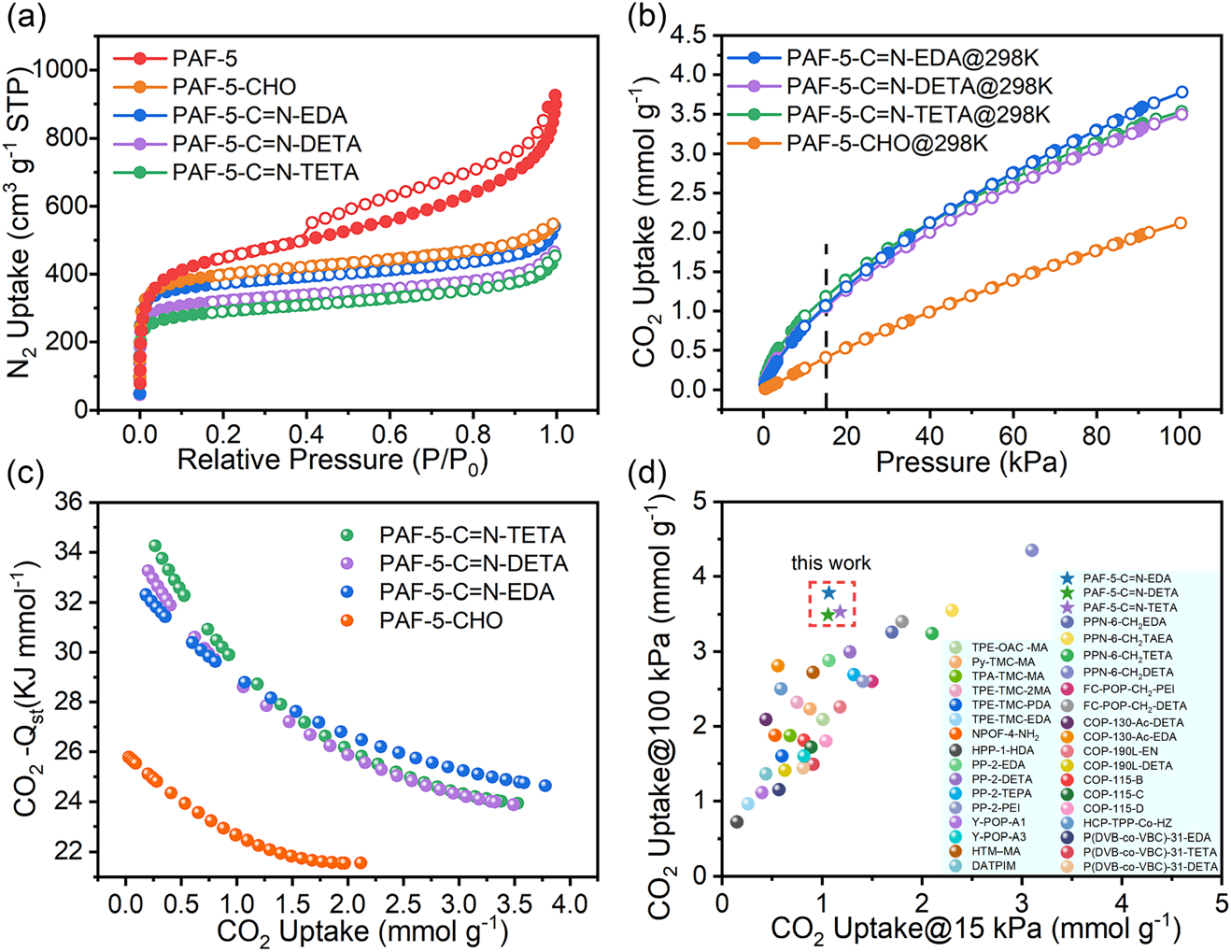
(a) N_2_ uptake at 77 K; (b) CO_2_ uptake of PAF-5-CHO and its derivations at 298 K; (c) CO_2_ −*Q*_st_ of PAF-5-CHO and its derivation; (d) comparison of CO_2_ uptake with other amine-functionalized POPs at 15 kPa and 100 kPa.^20,46–57^

The adsorption isotherms of PAF-5, PAF-5-CHO, PAF-5-C=N-EDA, PAF-5-C=N-DETA, and PAF-5-C=N-TETA at 77 K are shown in Fig. 3a. Their corresponding Brunauer–Emmett–Teller (BET) surface areas are 1660, 1510, 1423, 1224, and 1101 m^2^ g^−1^, respectively. The reduction in the BET surface area for PAF-5-CHO is likely due to the aldehyde groups occupying the pore channels, as evidenced by the decrease in pore width from 1.75 nm to 1.03 nm (Fig. S7†). PAF-5-C=N-EDA, PAF-5-C=N-DETA, and PAF-5-C=N-TETA exhibit further reductions in both BET surface area and pore volume relative to PAF-5-CHO. When the transformation of aldehyde groups into long-chains happens, the materials become heavier and the larger amine molecules occupy more pore space. Fig. 3b and S8† illustrate the CO_2_ adsorption isotherms of PAF-5-CHO and the three amine-functionalized PAF-5 materials at 298 K and 273 K. Across the entire experimental pressure range (0–100 kPa), the CO_2_ adsorption capacity of PAF-5-C=N-EDA, PAF-5-C=N-DETA, and PAF-5-C=N-TETA shows a remarkable improvement compared to PAF-5-CHO. This demonstrates that organic amine modification significantly enhances CO_2_ adsorption performance. Among the three materials, PAF-5-C=N-EDA shows the greatest improvement in CO_2_ adsorption capacity, primarily attributed to its high specific surface area and the high loading of amine. Specifically, at 298 K, the CO_2_ adsorption capacity of PAF-5-C=N-EDA is 3.78 mmol g^−1^, which represents a 78% increase compared to PAF-5-CHO, and the adsorption capacity remains nearly unchanged after six cycles (Fig. S9†).

Flue gas emitted from coal-fired power plants contains approximately 15% CO_2_ at 100 kPa, thus the CO_2_ adsorption capacity at 15 kPa is highly relevant for practical applications. At 298 K and 15 kPa, among the three amine-functionalized materials, PAF-5-C=N-TETA exhibits the highest CO_2_ adsorption capacity (Fig. S10†). Despite having the lowest BET surface area, PAF-5-C=N-TETA shows the highest nitrogen content—10.004% (Table S2†). This indicates that at low pressure, CO_2_ adsorption is more strongly influenced by the amine group content rather than the BET surface area. Specifically, at 298 K and 15 kPa, the CO_2_ adsorption capacity of PAF-5-CHO is 0.41 mmol g^−1^, while that of PAF-5-C=N-TETA reaches 1.18 mmol g^−1^, representing a twofold increase compared to PAF-5-CHO.

Furthermore, the isosteric enthalpy of adsorption (*Q*_st_) plot (Fig. 3c) determined from adsorption isotherm calculations at 273 K and 298 K shows that all three materials obtained after organic amine modification are significantly improved compared to PAF-5-CHO. The −*Q*_st_ of PAF-5-CHO is 25 kJ mol^−1^, whereas PAF-5-C=N-TETA, PAF-5-C=N-DETA, and PAF-5-C=N-EDA exhibit higher −*Q*_st_ values of 34, 33, and 32 kJ mol^−1^, respectively. Moreover, the CO_2_ desorption isothermal curves of these three materials do not exhibit significant hysteresis loops, indicating their excellent cycling potential. Compared to other amine-functionalized POPs (Fig. 3d), the three materials presented in this study exhibit higher adsorption capacities at both 15 kPa and 100 kPa. Thus, these materials demonstrate considerable promise for utilization in CO_2_ capture and recovery applications.

### Separation ability of PAF-C=N-EDA

**Fig. 4:**
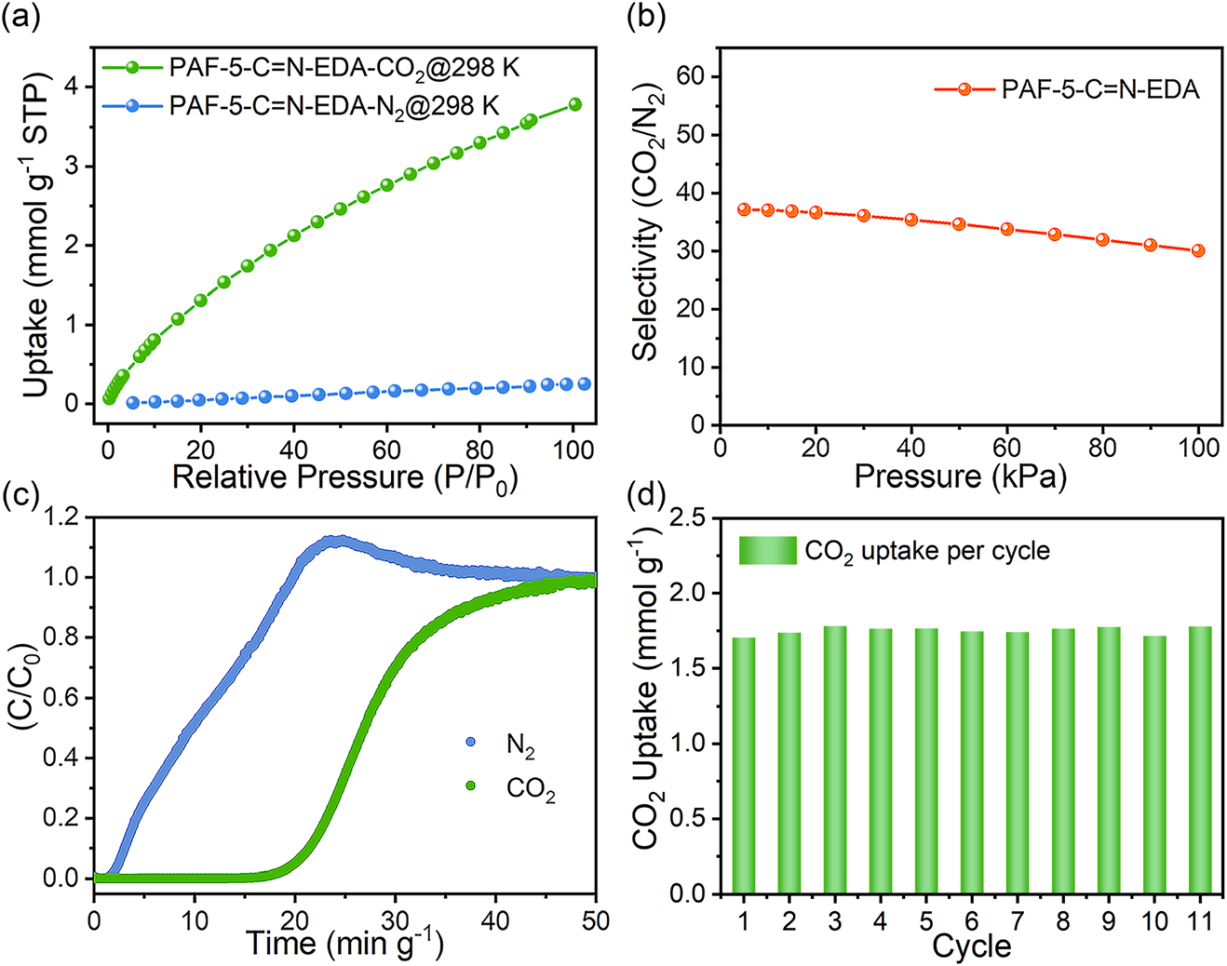
(a) N_2_ and CO_2_ uptake of PAF-5-C=N-EDA at 298 K; (b) CO_2_/N_2_ selectivity of PAF-5-C=N-EDA; (c) breakthrough curves of PAF-5-C=N-EDA for a CO_2_/N_2_ (15 : 85,v/v) gas mixture with a total flow of 10 mL min^−1^ at 298 K and 1 bar; (d) CO_2_ uptakes of PAF-5-C=N-EDA derived from the breakthrough cycling measurement.

Based on a comprehensive evaluation of the CO_2_ adsorption performance and the cost of the adsorbents, we selected PAF-5-C=N-EDA for subsequent CO_2_/N_2_ separation experiments. Using the CO_2_ and N_2_ adsorption isotherms of PAF-5-C=N-EDA at 298 K (Fig. 4a), the CO_2_/N_2_ selectivity was calculated using the Ideal Adsorbed Solution Theory (IAST) method. Fig. 4b shows that the CO_2_/N_2_ selectivity of PAF-5-C=N-EDA is approximately 37. To evaluate its actual separation performance, breakthrough experiments were conducted using 0.075 g of PAF-5-C=N-EDA powder. At 298 K and 1 bar, a CO_2_/N_2_ (15 : 85, v/v) gas mixture was passed through a tightly packed fixed-bed column containing PAF-5-C=N-EDA at a flow rate of 10 mL min^−1^. As shown in Fig. 4c, N_2_ breakthrough occurred almost immediately, while CO_2_ breakthrough was delayed for a retention time (15 min g^−1^), demonstrating excellent CO_2_ separation performance. The CO_2_ capture capacity calculated from a single breakthrough experiment was 1.00 mmol g^−1^, consistent with the results from the CO_2_ adsorption isotherm. After eleven consecutive breakthrough cycles (Fig. S11 and S12†), the material retained good separation performance, with an average CO_2_ adsorption capacity of 1.75 mmol g^−1^ during the breakthrough process.

### CO_2_ adsorption mechanism

To analyze the CO_2_ adsorption mechanism, we first performed density functional theory (DFT) simulations to investigate the potential interactions between the PAF-5-C=N-EDA framework and CO_2_ molecules. Five potential CO_2_ adsorption configurations were constructed and optimized (Fig. S13†). The calculated adsorption energies reveal that the interaction between the amine group and the C atom of CO_2_ represents the dominant contribution, exhibiting the lowest adsorption energy (−0.31 eV). This suggests that the amine groups serve as the most favorable active sites for CO_2_ adsorption. In this configuration, the N–C distance measures 2.76 Å, which is shorter than their sum of van der Waals radius. Additionally, the O–C–O angle of the adsorbed *CO_2_ deviates slightly from linearity, changing from 180° to 175.0°. Charge transfer analysis indicates electron donation from PAF-5-C=N-EDA to the adsorbed *CO_2_, with a Bader charge of 0.14|*e*| localized on *CO_2_. These results demonstrate that the adsorbed *CO_2_ undergoes activation, consistent with the observed negative adsorption energy. To further assess the diffusion rate of CO_2_ molecules on the PAF-5-C=N-EDA model, we examined the diffusion pathway and the corresponding energy barrier of a single CO_2_ molecule moving between two adjacent amine active sites (Fig. S14†). The computed diffusion barrier is remarkably low (0.09 eV), suggesting that CO_2_ molecules can diffuse rapidly through the porous structure of the material.

**Fig. 5:**
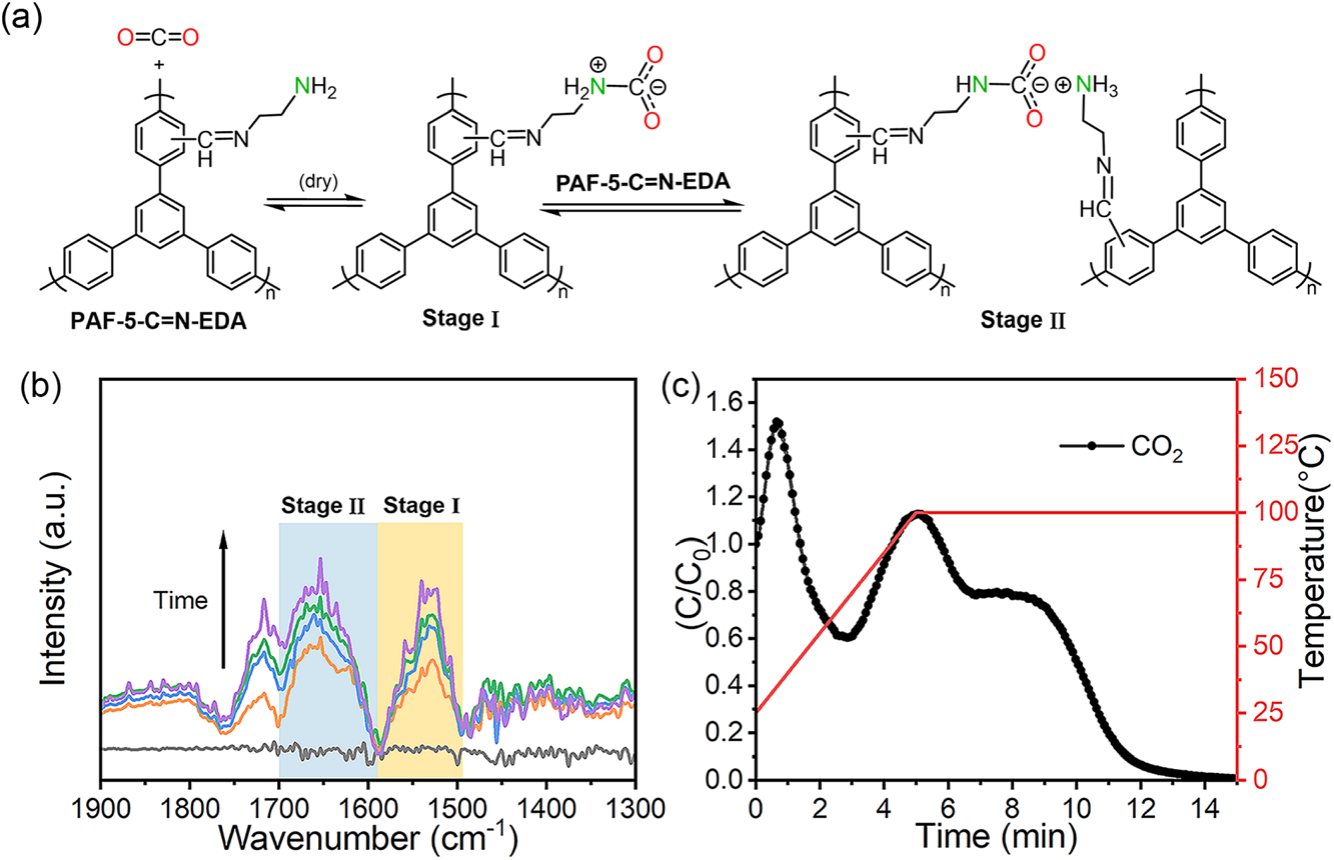
(a) Chemisorption mechanism of amines and CO_2_ under dry conditions; (b) IR absorption spectra of adsorbed species on PAF-5-C=N-EDA at 25 °C taken at different times during CO_2_ adsorption; (c) adsorbed phase analysis of PAF-5-C=N-EDA by using Temperature Programmed Desorption (TPD).

We conducted *in situ* IR spectroscopy to monitor the adsorption process of CO_2_ on PAF-5-C=N-EDA. During the experiment, CO_2_ gas flowed through the PAF-5-C=N-EDA adsorbent. The absorption spectra shown in Fig. S15† were obtained by collecting the PAF-5-C=N-EDA absorption spectra as a background, and the band produced by absorbed CO_2_ at 2360 cm^−1^ can be observed.^58^ In the IR spectra of PAF-5-C=N-EDA with adsorbed CO_2_ (Fig. 5b), the following features were identified: (i) the O=C=O^−^ band of carbamate NHCOO^−^ at 1532 cm^−1^ and (ii) the ^+^N–H band of NH_3_^+^ at 1656 cm^−1^. The adsorption mechanism inferred from the *in situ* IR analysis is illustrated in Fig. 5a, and the process occurs in two stages. To further confirm the adsorption mechanism, temperature-programmed desorption (TPD) of the post-breakthrough sample was performed (Fig. 5c). The first desorption peak corresponds to weakly adsorbed CO_2_ on the adsorbent and the column walls, while the second peak, appearing at temperatures above 100 °C, corresponds to the intermediate species described as Stage I in the mechanism diagram. A third peak represents the desorption of CO_2_ species associated with Stage II of the mechanism temperatures above 100 °C, corresponding to the intermediate species described as Stage I in the mechanism diagram. A third peak represents the desorption of CO_2_ species associated with Stage II of the mechanism.

## Conclusion

We reported a post-modification method for PAFs that can introduce aldehyde groups into the framework of PAF-5 in a simple and efficient manner. Materials modified with aldehyde groups can be subjected to the subsequent reactions, and in this paper, we prepared a series of amino-modified PAFs by using the aldehyde group for the Schiff base reaction. Such materials were characterized using CO_2_ adsorption–desorption isotherms and CO_2_/N_2_ dynamic breakthroughs, and we observed that PAF-5-C=N-EDA exhibits excellent performance in capturing post-combustion CO_2_. Through the investigation of the CO_2_ adsorption mechanism, it was found that there are two distinct adsorption sites involved in the process. This study highlights the potential of this class of materials in post-combustion CO_2_ capture technology.

## Experimental

### Materials

All starting materials and solvents, unless otherwise specified, were obtained from Innochem Co. and used.

### Synthesis of PAF-5 as reported

In a glovebox, 1,5-cyclooctadiene (cod) (0.93 mL, 7.92 mmol), bis(1,5-cyclooctadiene) nickel (0) (Ni(cod)_2_) (2.18 g, 7.93 mmol), 2,2′-bipyridyl (1.24 g, 7.94 mmol), and anhydrous *N*,*N*′-dimethylformamide (DMF) (40 mL) were added to a 250 mL double-neck flask. A solution of 1,3,5-tris(4-bromophenyl) benzene (TBB) in DMF (80 mL) was then slowly added to the mixture at 80 °C. The resultant system was further kept at 80 °C for 48 h. After cooling to room temperature, 6 M HCl was added to the mixture, and the mixture was stirred for 1 hour until it turned green with a snowflake-like solid formation, washed and dried to obtain the product.

### Synthesis of PAF-5-CHO

PAF-5 (100 mg), anhydrous AlCl_3_ (200 mg, 1.5 mmol) and CHCl_3_ (40 mL) were added to a 100 mL flask. The mixture was stirred vigorously, heated to 80 °C and the reaction continued for one day. After quenching with 50 mL of methanol, the solid was filtered, and washed using 1 M hydrochloric acid and water successively. After drying under vacuum, a brownish yellow solid was obtained.

### General procedure for the amine-functionalized materials

Take the synthesis of PAF-5-C=N-EDA as an example: PAF-5-C=N-EDA (100 mg) and ethylenediamine (EDA, 10 mL) were added to a sealed flask. The mixture was kept at 80 °C for 3 days. The solid was filtered, washed with water and methanol, and then dried *in vacuo* to produce PAF-5-C=N-EDA as a brown powder in quantitative yield.

### Materials and physical measurements

Fourier transform infrared spectroscopy (FTIR) spectra were recorded using a Nicolet IS50 Fourier transform infrared spectrometer. Solid-state ^13^C cross-polarization magic angle spinning nuclear magnetic resonance (NMR) spectra were acquired with a Bruker Avance III 400 MHz NMR spectrometer at a MAS rate of 5 kHz. X-ray photoelectron spectroscopy (XPS) spectra were obtained on a KRATOS Axis Ultra DLD system, equipped with an Al Kα ray source (*hv* = 1486.6 eV) under a high vacuum of 9.8 × 10^−10^ torr. N_2_ isotherms at 77 K and vapor isotherms were measured using an Autosorb adsorptometer (JW-TB 400 and JW-ZQ 100). Thermal gravimetric analysis (TGA) was performed on a METTLER-TOLEDO TGA/DSC 3+ analyzer at a heating rate of 10 °C min^−1^ under an air flow of 60 mL min^−1^. Scanning electron microscope (SEM) images were captured using a Hitachi SU-70 microscope. Powder X-ray diffraction (PXRD) was performed on a Rigaku Smartlab X-ray diffractometer with Cu-Kα radiation at 40 kV and 200 mA with a step size of 0.01. Elemental analysis was carried out with a Eurovector EA3000 Analyzer. Elemental oxygen analysis was carried out with a Vario EL cube.

### Breakthrough experiments

Breakthrough experiments were performed using a dynamic gas breakthrough device, mixSorb S (3P Instruments). The sample was heated under a vacuum at 100 °C for 8 h. A 0.075 g sample of PAF-5-C=N-EDA was loaded onto a stainless-steel column (1 mL volume, 0.45 cm inner diameter). A CO_2_/N_2_ (15: 85, v/v) gas mixture was allowed to flow into the column. A mass spectrometer (Master 400) was used to monitor the gas flow from the column.

## Author contributions

J. J., G. Z., and Z. B. conceptualized and supervised. Q. W. planned research and synthesis; Q. W. and L. L. analysed the data. Q. W. and L. L. wrote the manuscript. L. J., Z. W., and Y. Z. synthesized and analysed for the discussion. Q. H. performed SEM characterization, and J. J. and G. Z. performed the revision.

## Conflicts of interest

There are no conflicts to declare.

## Supplementary Material

SC-016-D5SC00355E-s001

## Data Availability

The data are available in the ESI.†
