# Supplementary material for: A new post-synthetic route to graft amino groups in porous organic polymers for CO2 capture
Source: Chem Sci. 2025 Jul 1;16(33):15121–8. doi: 10.1039/d5sc00355e (PMC12282537; doi:10.1039/d5sc00355e)
Supplement: SC-016-D5SC00355E-s001 [file SC-016-D5SC00355E-s001.pdf]

Supplementary Information (SI) for Chemical Science.
This journal is © The Royal Society of Chemistry 2025

Electronic Supplementary Information (ESI) :

A new post-synthetic route to graft amino groups in porous organic polymers for CO_2 capture

Qihaoyue Wang [a+], Lin Lin [a+], Li Jiang [a], Zihao Wang [a], Yina Zhang [a], Qiance Han [a], Xin Huang [a],Changyan Zhu [a] , Jiangtao Jia [a*], Zheng Bian [a*], Guangshan Zhu [a*]

a. Key Laboratory of Polyoxometalate and Reticular Material Chemistry of Ministry of Education, Faculty of Chemistry, Northeast Normal University, Changchun, 130024, Jilin, China. jiangtaojia@nenu.edu.cn; bianz070@nenu.edu.cn; zhugs@nenu.edu.cn

b. † Q. Wang and L. Lin contribute equally to this paper.

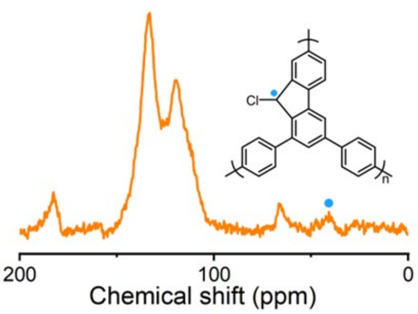

Figure S1 Schematic illustration of the product corresponding to the 40 ppm signal in ^{13}C CP/MAS NMR spectrum

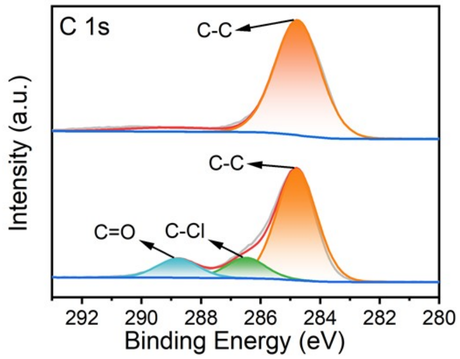

Figure S2 XPS C 1s spectrum of PAF-5 and PAF-5-CHO.

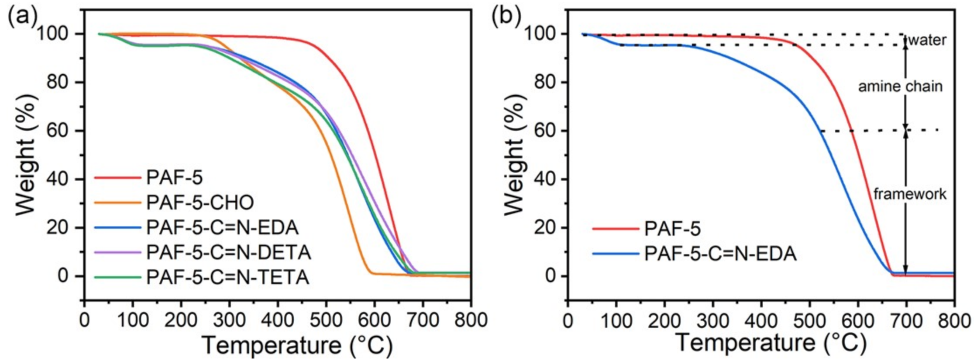

Figure S3 (a) Thermogravimetric traces of PAF-5, PAF-5, PAF-5-CHO, PAF-5-C=N-EDA, PAF-5-C=N-DETA and PAF-5-C=N-TETA under air flow; (b) Thermogravimetric data of PAF-5-C=N-EDA with proposed weight loss.

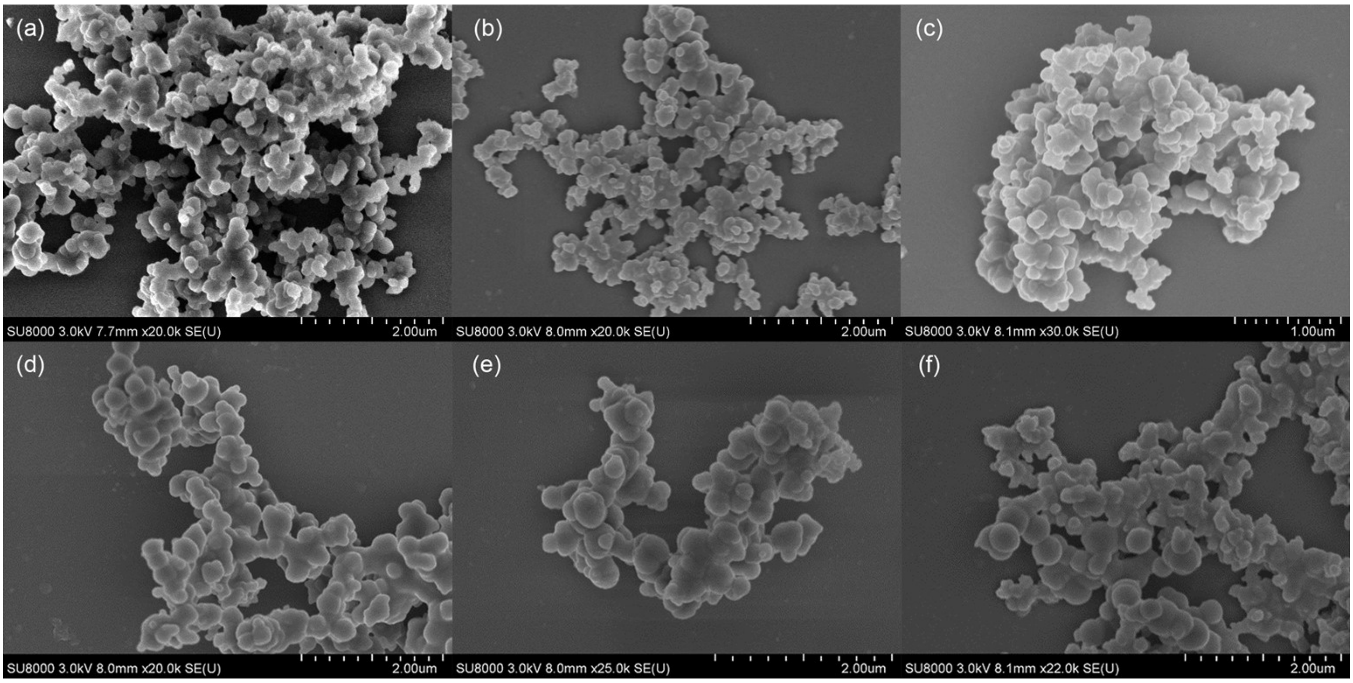

Figure S4 Morphology of (a) PAF-5, (b and c) PAF-5-CHO, (d) PAF-5-C=N-EDA, (e) PAF-5-C=N-DETA and (f) PAF-5-C=N-TETA by SEM.

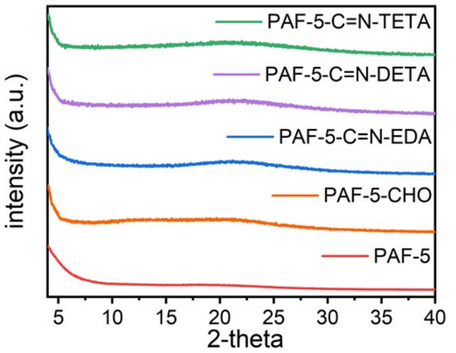

Figure S5 PXRD patterns of PAF-5 and a range of its post-modified materials.

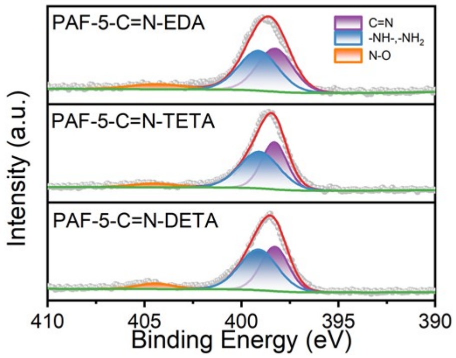

Figure S6 XPS C 1s spectrum of PAF-5 and PAF-5-CHO.

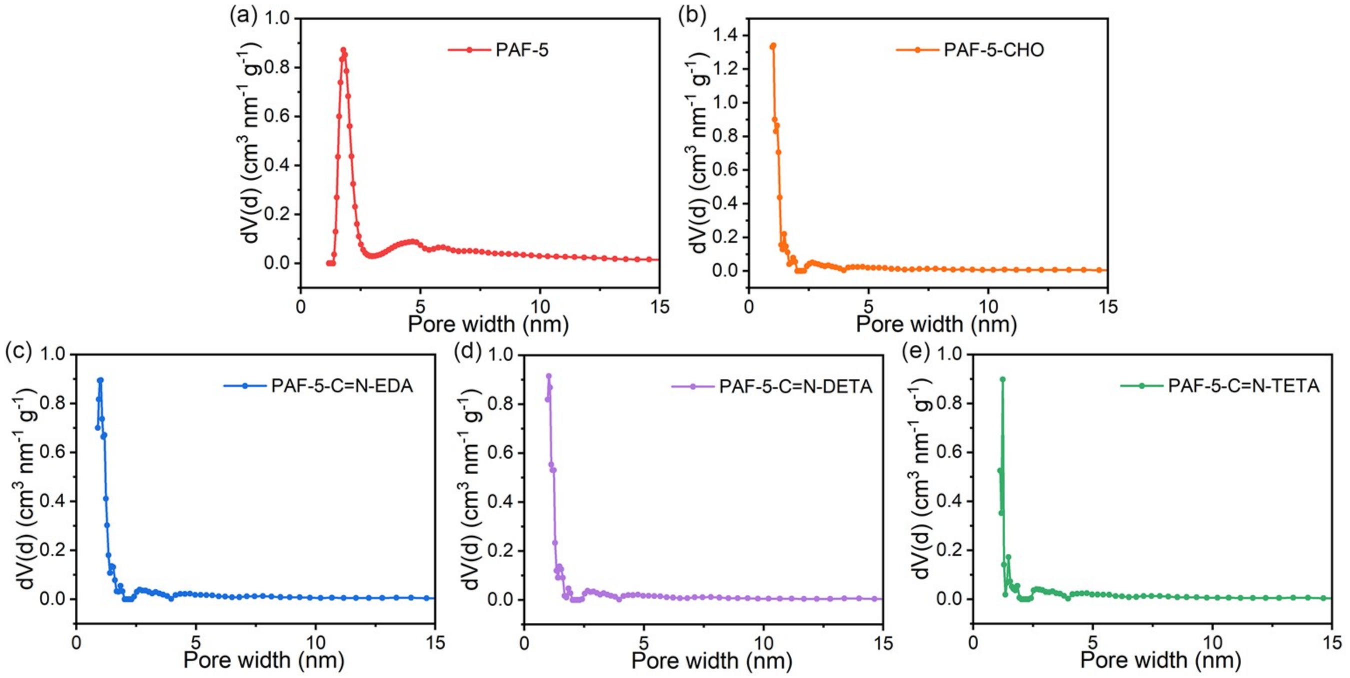

Figure S7 The pore size distributions of PAF-5 (a), PAF-5-CHO (b), PAF-5-C=N-EDA (c), PAF-5-C=N-DETA(d) and PAF-5-C=N-TETA were determined. Fitting the isotherm based on NLDFT revealed a consistent pore size distribution characterized by a narrow peak at 1.42 nm for PAF-5, 1.03 nm for PAF-5-CHO, 1.00 for PAF-5-C=N-EDA, 1.05 for PAF-5-C=N-DETA and 1.23 nm for PAF-C=N-TETA.

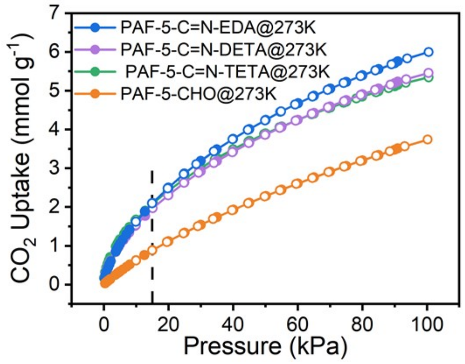

Figure S8 CO_2 uptake of PAF-5-CHO and its derivations at 273 K.

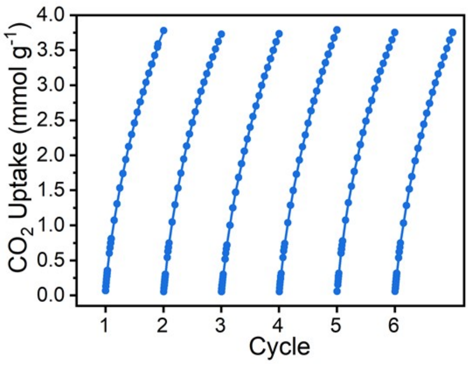

Figure S9 CO_2 cycles adsorption of PAF-5-C=N-EDA.

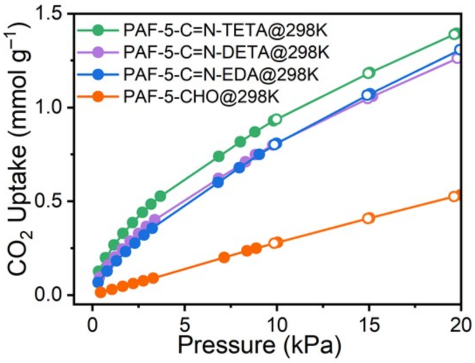

Figure S10 Magnified view of the CO_2 sorption isotherm taken from a highlighting the uptake at the CO_2 pressure (0-20 kPa).

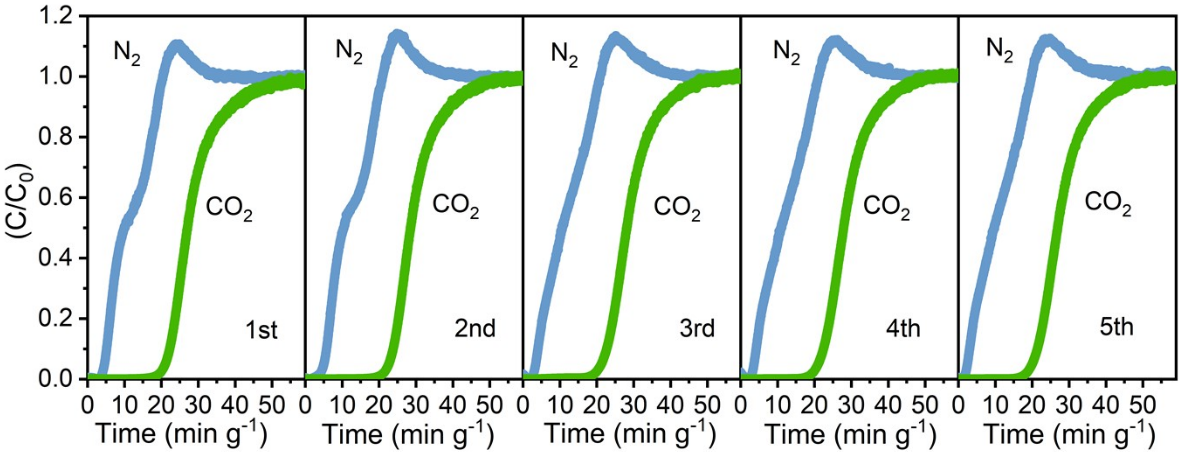

Figure S11 Dynamic breakthrough cycle curves for CO_2/N_2 (15/85) mixtures at 25 °C (1st-5th).

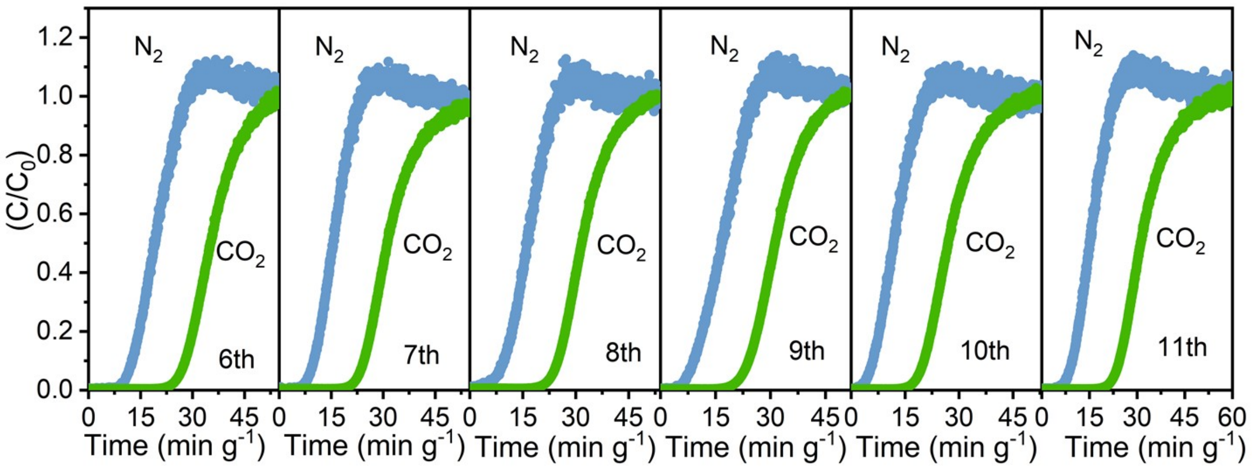

Figure S12 Dynamic breakthrough cycle curves for CO_2/N_2 (15/85) mixtures at 25 °C (6th-11th).

Computation Method

Density functional theory (DFT) [1] simulations were performed with the Vienna ab initio simulation package (VASP) [2]. The Perdew-Burke-Ernzerhof (PBE) functional within the generalized gradient approximation (GGA) and the projector augmented-wave (PAW) potential were employed [3]. The PAF-5-C=N-EDA model was constructed with three monolayers, including 171 C atoms, 18 N atoms and 144

H atoms. The plane-wave cutoff energy of 500 eV and the Monkhorst-Pack k-points mesh of 1 × 1 × 1 were adopted for all computations. The convergence criteria were set at 10^{-5} eV for total energy change and 0.05 eV Å-1 for the maximum forces on each atom, respectively. The Grimme's semiempirical DFT-D3 method of dispersion correction was included to properly describe the van der Waals (vdW) interactions [4]. The adsorption energy of CO_2 were calculated by ΔEad = E*CO2 – E* - ECO2, where E*CO2 and E* is the energy of PAF-5-C=N-EDA model with and without CO_2; ECO2 is the energy of CO_2 molecule.

To investigate the CO_2 diffusion energy barrier, the climbing-image nudged elastic band (CI-NEB) method is used to search the minimum energy pathway between the given initial and final configurations [5].

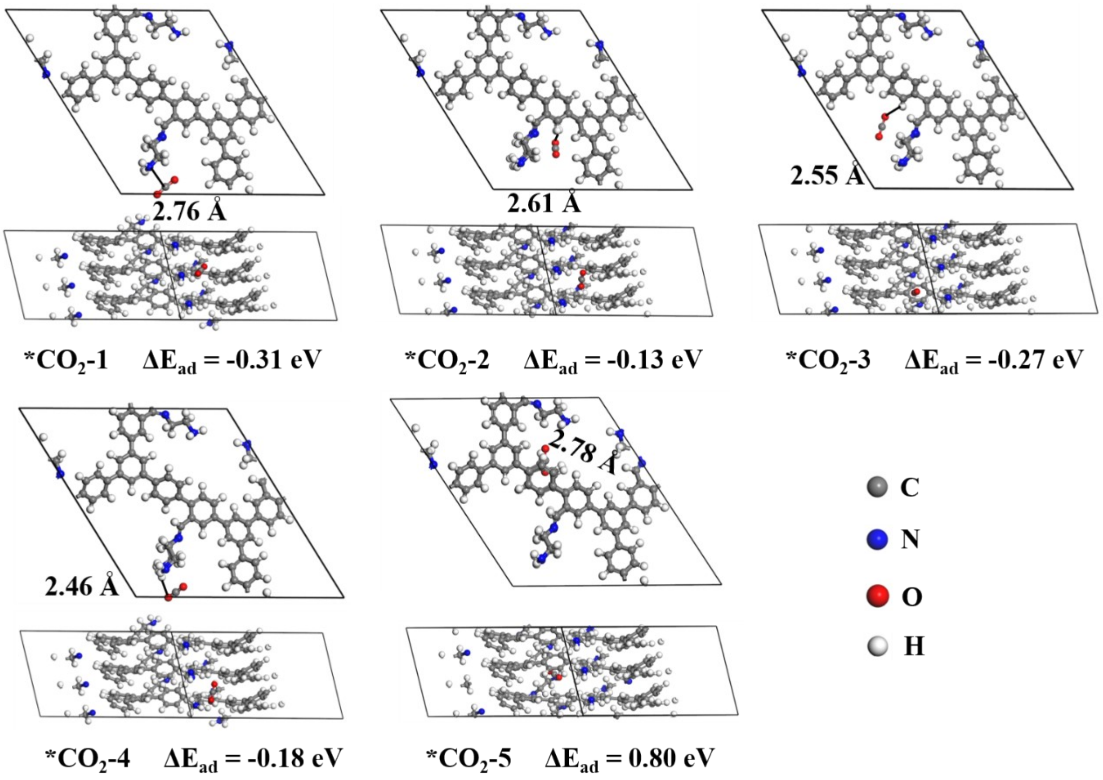

Figure S13. Five potential configurations of CO_2 adsorbed on the PAF-5-C=N-EDA model and the corresponding adsorption energies.

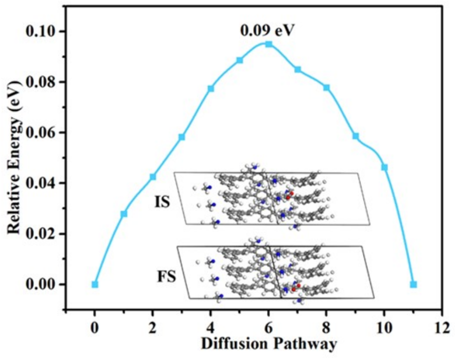

Figure S14. The diffusion barrier and diffusion pathway of CO_2 molecules on the PAF-5-C=N-EDA model.

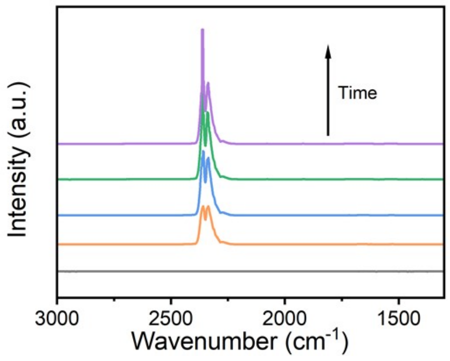

Figure S15 IR absorbance spectra of CO_2 adsorption on PAF-5-C=N-EDA at different times.

Table S1. Elemental Oxygen Analysis data

Name	Weight (mg)	O%
Benzoic acid	4.6630	26.200
PAF-5-CHO	1.9400	7.475

Table S2. Elemental Analysis data

	PAF-5-CHO	PAF-5-C=N-TETA	PAF-5-C=N-DETA	PAF-5-C=N-EDA
N%	0.1	10.044	9.438	9.278
C%	67.144	69.387	70.786	71.596
H%	2.796	5.403	5.184	5.403

Table S3. Porosity of amino-functionalized PAF-5-CHO series materials

	BET surface area （m^2 g^{-1}）	Pore volume （cm^3 g^{-1}）	Pore width (nm)
**PAF-5**	1660	1.391	1.75
**PAF-5-CHO**	1510	0.085	1.03
**PAF-5-C=N-EDA**	1423	0.084	1.00
**PAF-5-C=N-DETA**	1224	0.072	1.05
**PAF-5-C=N-TETA**	1101	0.070	1.23

[1] D. Singh, J. Ashkenazi, *Phys. Rev. B* 1992, *46*, 11570.
[2] B. Barbiellini, M. Puska, T. Korhonen, A. Harju, T. Torsti, R. Nieminen, *Phys. Rev. B* 1996, *53*, 16201.
[3] a) P. Blöchl, *Phys. Rev. B* 1994, *50*, 17953; b) G. Kresse, G. Joubert, *Phys. Rev. B* 1999, *59*, 1758.
[4] S. Grimme, *J. Comput. Chem.* 2006, *27*, 1787.
[5] G. Mills and H. Jónsson, *Phys. Rev. Lett.*, 1994, 72, 1124.
